# Population Genomics of Inversion Polymorphisms in *Drosophila melanogaster*

**DOI:** 10.1371/journal.pgen.1003056

**Published:** 2012-12-20

**Authors:** Russell B. Corbett-Detig, Daniel L. Hartl

**Affiliations:** Department of Organismal and Evolutionary Biology, Harvard University, Cambridge, Massachusetts, United States of America; Fred Hutchinson Cancer Research Center, United States of America

## Abstract

Chromosomal inversions have been an enduring interest of population geneticists since their discovery in *Drosophila melanogaster*. Numerous lines of evidence suggest powerful selective pressures govern the distributions of polymorphic inversions, and these observations have spurred the development of many explanatory models. However, due to a paucity of nucleotide data, little progress has been made towards investigating selective hypotheses or towards inferring the genealogical histories of inversions, which can inform models of inversion evolution and suggest selective mechanisms. Here, we utilize population genomic data to address persisting gaps in our knowledge of *D. melanogaster'*s inversions. We develop a method, termed Reference-Assisted Reassembly, to assemble unbiased, highly accurate sequences near inversion breakpoints, which we use to estimate the age and the geographic origins of polymorphic inversions. We find that inversions are young, and most are African in origin, which is consistent with the demography of the species. The data suggest that inversions interact with polymorphism not only in breakpoint regions but also chromosome-wide. Inversions remain differentiated at low levels from standard haplotypes even in regions that are distant from breakpoints. Although genetic exchange appears fairly extensive, we identify numerous regions that are qualitatively consistent with selective hypotheses. Finally, we show that *In(1)Be*, which we estimate to be ∼60 years old (95% CI 5.9 to 372.8 years), has likely achieved high frequency via sex-ratio segregation distortion in males. With deeper sampling, it will be possible to build on our inferences of inversion histories to rigorously test selective models—particularly those that postulate that inversions achieve a selective advantage through the maintenance of co-adapted allele complexes.

## Author Summary

Chromosomal inversions are known to respond to powerful natural selection in many species. Despite this evidence, little progress has been made towards understanding the nature of selection that affects inversions. Here, we utilize two recently released population-resequencing projects from *D. melanogaster* to address many of the unknown features of polymorphic inversions. We find evidence that inversions in this species are generally very young, with ages on the order of hundreds to tens of thousands of years, and that the majority of inversions originated in ancestral African populations. Inversions are also the source of the majority of genetic structure within populations and affect polymorphism chromosome-wide. We are able to confirm experimentally that one X-chromosome inversion achieves an advantage by selfishly increasing its transmission through males. Future work will build on our basic inferences to identify potential selective mechanisms and candidate genes in the other inversions studied.

## Introduction

Since their initial discovery in *Drosophila melanogaster* [1], chromosomal inversions have been the topic of many analyses and much speculation. A growing body of literature suggests that inversions may play a role in speciation [2], [3], local adaptation [4], and the maintenance of segregation distortion complexes [5]–[7], among other potential selective mechanisms (reviewed in [4], [8]). Empirical surveys indicate that inversions are pervasive, and polymorphic inversions have been indentified in virtually all species that have been carefully scrutinized [8], [9]. In many species, including plants, fungi, insects and humans, there is evidence that inversions respond to natural selection; however few genes or other chromosomal features that are the targets of selection have been unambiguously identified. Thus, the mechanisms of selection that affect most inversions remain unknown [8].

Owing to its position as a premier model and the facility with which inversions can be assayed cytologically, the *Drosophila* genus has been a favored system for studying polymorphic inversions in natural populations [10]. Nearly a century of work has yielded numerous lines of evidence that suggest strong selection governs the distributions of inversions in these species. Much of the earliest data consistent with selection on inversions was obtained from *D. pseudoobscura* (reviewed in [10], [11]). Although our analysis and discussion will focus on data from *D. melanogaster*, the patterns we observe may represent general phenomena and are consistent with evidence that has accumulated in a variety of other species [8]. Frequency clines of the most common *D. melanogaster* inversions are independently replicated on many continents, and quickly reestablish following colonization events [12]–[15]. Recurrent seasonal frequency shifts have been observed in numerous geographically diverse populations [14], [16]. Finally, heterozygote superiority has been reported in both laboratory and natural populations [12], [17], [18]. Collectively, these findings suggest powerful selective mechanisms affect the distributions of polymorphic inversions in *D. melanogaster*.

Despite continuing efforts, many unaddressed gaps remain in our understanding of the inversion polymorphisms of *D. melanogaster*. First, the breakpoints of only three inversions have been examined at the nucleotide level [19]–[21]. Second, largely due to a paucity of nucleotide data, few attempts have been made towards estimating the genealogical histories of polymorphic inversions, which may suggest selective mechanisms and inform tests of selective hypotheses (although see [19]–[22]). Third, we have little data on the degree to which inversions affect polymorphism throughout chromosome arms. Finally, the selective pressures that affect the distributions of inversions in *D. melanogaster* have rarely been identified conclusively, with notable exceptions being inversions associated with the *Segregation distortion* complex [5], [7].

Recently, Corbett-Detig *et al.* [23] developed a method of inversion breakpoint detection based on next-generation sequence data, and they applied this method to a large sample of African *D. melanogaster* genomes. In total, they identified eight polymorphic inversions in African and Cosmopolitan populations of *D. melanogaster*. Four inversions, termed “common cosmopolitan”, have been recovered in almost all populations worldwide [10]. These inversions have been the subjects of most population frequency assays and fitness assays in this species. Corbett-Detig *et al.* [23] also recovered two “rare cosmopolitan” inversions, *In(3R)Mo* and *In(3R)K*, and two “recurrent endemic” inversions that are only known from African populations, *In(1)A* and *In(1)Be* (Table 1) [24]. Little work has focused on these rare cosmopolitan and endemic inversions, which are expected to be relatively young and therefore may provide information about the selective pressures that affects an inversion's initial rise in frequency.

**Table 1:** Summary information for the inversion breakpoints studied.

Inversion	Classification	Distribution	Breakpoint	Position
In(2L)t	Common Cosmopolitan	High frequencies worldwide	Distal	2225744
			Proximal	13154180
In(2R)NS	Common Cosmopolitan	High frequencies worldwide	Proximal	11278659
			Distal	16163839
In(3R)K	Rare Cosmopolitan	High frequency in Africa, rare elsewhere	Proximal	7576289
			Distal	21966092
In(3R)Mo	Rare Cosmopolitan	Rare worldwide, absent in Africa	Proximal	17232639
			Distal	24857019
In(3R)P	Common Cosmopolitan	High frequencies worldwide	Proximal	12257931
			Distal	20569732
In(3L)P	Common Cosmopolitan	High frequencies worldwide	Distal	3173046
			Proximal	16301941
In(1)A	Recurrent Endemic	Rare in Africa	Distal	13519769
			Proximal	19473361
In(1)Be	Recurrent Endemic	Rare in Africa	Distal	17722945
			Proximal	19487744

Here, we use these new tools in combination with data from two publically available *D. melanogaster* sequencing datasets [25], [26] to investigate the genealogical histories of polymorphic inversions in these populations. Consistent with the demographic history of this species [25], [27], [28], and previous work on inversions in this species [19], [20], [22], our data support a recent African origin for most inversions. We examine the effects of these inversions on polymorphism throughout the genome as well as the selective models proposed to explain the initial rise in frequency and maintenance of inversions in natural populations; we find numerous examples that are qualitatively consistent with selection. Finally, conspicuous population genetic signatures suggest, and we confirm experimentally, that one X-chromosome inversion achieves a transmission advantage via sex-ratio distortion. In combination with deeper sampling, especially in ancestral African populations, it will be possible to build on our genealogical inferences to test a range of selective models in this species.

## Results/Discussion

### Reference-Assisted Reassembly (RAR)

Because they remain in strong linkage disequilibrium with inversions, sequences surrounding breakpoints are often used as a means of investigating inversion genealogical histories [*e.g.* 19], [20], [22]. However, the utility with which standard genome assemblies can be used to estimate true levels of polymorphism is questionable. Pool *et al.* [25] note a strong correlation between sequencing depth and divergence from the reference sequence in the dataset produced by the second sequencing phase of the *Drosophila* population genomics project (DPGP2). They attribute this to reference bias, or the inherent ascertainment bias against non-reference alleles. It is straightforward to imagine that systematically underestimating polymorphism may downwardly bias estimates of the time since recent common ancestry. To mitigate this potential bias, we developed RAR. Briefly, this method works by aligning all reads to a reference sequence, and subsequently parsing reads and their pairs from particular genomic regions and *de novo* reassembling this set. This enables RAR to recruit reads that are not initially mapped to the reference, provided their paired-end does, and to resolve highly polymorphic regions including insertion and deletion polymorphisms.

The interpretation of consensus quality is an unresolved issue in population genomics. Due to the additional complication of a *de novo* reassembly step and the difficulty of simulating data that accurately reflect true patterns of polymorphism, we favor an empirical confirmation of RAR consensus quality. Three of the strains sequenced as a part of DPGP2 have been studied extensively for PCR-based demographic analyses in this species [27], [29]. To estimate the error rate of RAR, we downloaded more than 50 kb of PCR sequence data for each strain (See Supplemental Table 1 for EMBL ascension numbers), and used RAR to rebuild the corresponding regions from next-generation short-read data. The majority of these sequences are derived from intergenic regions in one of the most diverse populations of *D. melanogaster* [25]. These sequences are therefore a conservatively challenging test of RAR's performance. In total, we identified 23 single-nucleotide mismatches between Sanger-PCR fragments and corresponding RAR sequences. After resequencing via PCR all fragments that contained a mismatch (Supplemental Dataset S1), we found that all discrepant sites matched the RAR consensuses. Thus, the point estimate for RAR's error rate is 0. Assuming errors in the RAR assemblies are Poisson distributed, the upper 95% confidence interval of RAR's error rate corresponds to three errors, or approximately Q47.

**Figure 1:**
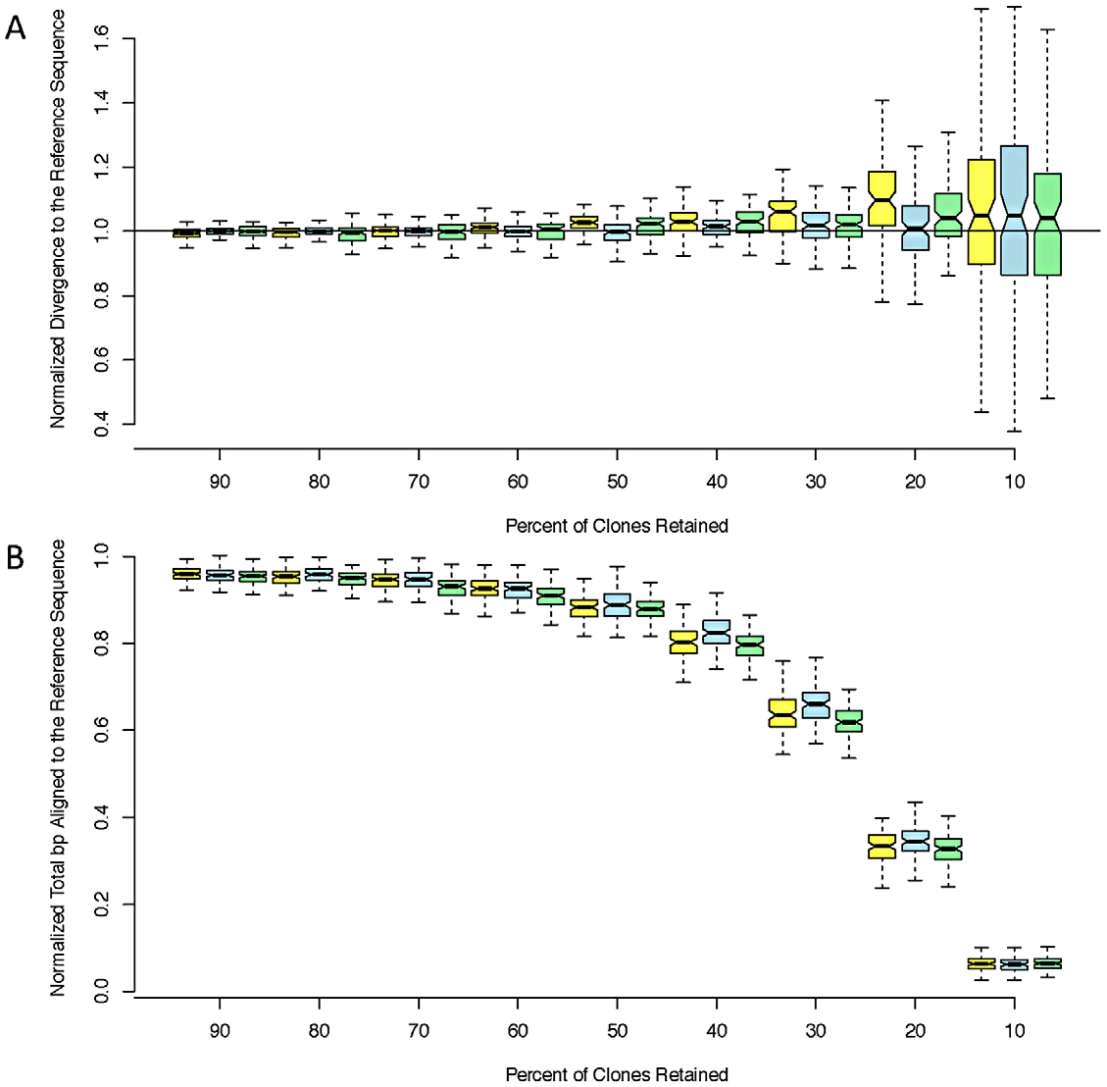
Estimated divergence. Estimated divergence to the reference (A) and coverage (B) at decreasing read depths for three strains: ZK131 (yellow), ZK186 (blue), and ZK84 (green). We normalized both coverage and divergence by dividing by the ‘true’ values obtained from the aligned PCR sequences. Each box corresponds to 100 bootstrap replicates.

While error rates are of interest, the most important and direct consequence of reference bias for population genetic inference is decreased polymorphism in resequenced individuals relative to the reference genome. In particular, reference bias is exacerbated by shallow sequencing depth [25]. We found that the RAR consensus sequences yielded nearly identical estimates of divergence to the reference genome as the Sanger-PCR sequences. The corresponding sequences produced by Pool et al. [25] using BWA and samtools [30], [31] underestimate divergence from the reference by approximately 25% and align fewer bases (Table 2). To investigate the effect of sequencing depth on RAR's performance, we performed bootstrap replicates by discarding read pairs at random. RAR is robust to decreased sequencing depth, and can produce accurate, unbiased assemblies even with only 10% of reads retained (∼2× depth; Figure 1).

**Table 2:** Comparison of divergence to the reference sequence calculated using sanger PCR sequences, RAR, and the assemblies produced by Pool *et al*. [25].

Line	Assembly	Bases Aligned	SNPs	π
ZK84	PCR	52099	568	0.0109
	RAR	51456	557	0.0108
	BWA DPGP2	48553	398	0.0082
ZK131	PCR	52134	561	0.0108
	RAR	51540	553	0.0107
	BWA DPGP2	49060	422	0.0086
ZK186	PCR	51985	642	0.0123
	RAR	51300	636	0.0124
	BWA DPGP2	48454	433	0.0089

It is important to note that reference bias may persist in RAR assemblies. To whatever degree sequencing biases, such as biases in GC composition, are correlated with true patterns of genomic variation is an important potential confounding factor. That there are few models of these and related biases precludes an in-depth examination of this problem. Nonetheless, we do not observe any indirect effects of these or related biases in our validation, suggesting that RAR will be sufficient for our analyses and may be widely serviceable for a variety of other applications.

### Divergence-Based Age Estimates

We used RAR to rebuild intergenic regions immediately inside inversion breakpoints, which are expected to reflect the genealogical histories of inversions due to strongly suppressed recombination with the standard arrangement. Prior to all subsequent analyses, we removed sequences that appeared to result from exchange between arrangements (see methods).

If an inversion has fixed nucleotide substitutions since its formation, the time to the most recent common ancestor (*T_MRCA_*) is an underestimate of the true time since formation. This may be especially likely if inversions are prone to hitchhiking effects [32] due to reduced recombination. A divergence-based metric (*e.g.* [22]), may be an appealing alternative for estimating the age of inversions. We calculated pairwise divergence (π) at breakpoint regions between inverted and standard haplotypes and between all standard haplotypes. Subtracting π among standard haplotypes from π between inverted and standard haplotypes and subsequently normalizing by the local mutation rate yields an estimate of the time since formation of an inversion [22]. At most breakpoints studied, this quantity is very small (or negative; Table S2), which suggests that inversions are very recently derived from the standard arrangement. Importantly, this is unlikely to stem from a bioinformatic artifact as we have taken strong precautions against underestimating divergence to a standard-arrangement reference haplotype.

Hasson and Eanes [22] found that *In(3L)P* is considerably more divergent from standard haplotypes than we estimated (Table S1). The contrast with our results likely stems from the differences in the selection of standard strains for comparison. As a result of a recent bottleneck, cosmopolitan populations have significantly decreased polymorphism relative to ancestral populations [25], [33]. In the analysis of [22], all but one of the standard strains are derived from cosmopolitan populations. This could cause the sampled standard sequences to be more closely related, to the exclusion of cosmopolitan inverted haplotypes, with which exchange is suppressed, than if standard strains had been selected from a diverse African population. When we recalculated the same divergence-based estimate using the standard French haplotypes in the DPGP2 dataset and all inversion-bearing haplotypes, the inverted haplotypes appear much more differentiated from the standard sequences, and our estimate (340,000 years) is consistent with that of ref. [22].

The lack of genetic divergence between arrangements does not necessarily indicate a recent origin of inversions, as this pattern may also result from a combination of genetic exchange and occasional selective sweeps, which periodically eliminate variation, causing inversions to appear more recently derived from a standard haplotype than they are in actuality. We cannot formally exclude this explanation; however recent data from 3^rd^ chromsome inversions of *D. pseudoobscura* [34] demonstrate that sequences near inversion breakpoint harbor more polymorphism than segments more distant from breakpoints and collinear regions of the genome. While it is possible that the two species differ in some other fundamental biological feature (e.g. the mechanism or rates of recombination in heterokaryotypes, the frequency of selective sweeps, etc.), a simpler explanation is that the inversions of *D. melanogaster* have a more recent origin than those of *D. psuedoobscura*. The data from *D. pseudoobscura* therefore support the use of age estimates based on polymorphism among inverted haplotypes.

### Polymorphism-Based Age Estimates

Both inverted and standard allele frequency spectra, summarized as Tajima's *D* [35] and *D′* [36], are skewed towards rare alleles (Table S3). This skew is consistent with demographic models for the species that suggest a recent population expansion in African populations of *D. melanogaster* [27], a recent range expansion, or pervasive selection [25]. However, inversion *D′* values tend to be more negative than corresponding standard arrangements. In one case, *In(1)Be*, there are no segregating sites present on inverted haplotypes (Table S3). Given this excess of rare alleles, and paucity of polymorphisms, it is reasonable to suppose that most inversions have only recently achieved their present frequencies.

Because sequences tightly linked to an inversion breakpoint effectively create a single rarely-recombining “locus”, it is not feasible to fit complex models to these data. Instead, we assume a simple model of exponential growth from the time of inversion formation through the present. This approach may be preferable to the minimum age estimate described in [19], as it does not assume neutrality and demographic equilibrium of the population or an explicit effective population size. In addition, it is possible using our approach to quantify the variance of our estimate via an ABC method [28], [37], [38]. Although there are many advantages to our approach, we stress that these results should be interpreted cautiously because any simple model is unlikely to reflect an inversion's true history. In the case of *In(2R)NS*'s proximal breakpoint, we obtained a very low acceptance rate during the ABC run (1.52*10^−5^), suggesting that the model may be a poor fit for this sequence. Other breakpoints' acceptance rates are at least one order of magnitude greater. While undoubtedly the age estimates are approximations, they should be sufficient for comparative purposes, and in the case of younger inversions, this model is likely a reasonable facsimile of the inversion's history.

**Figure 2:**
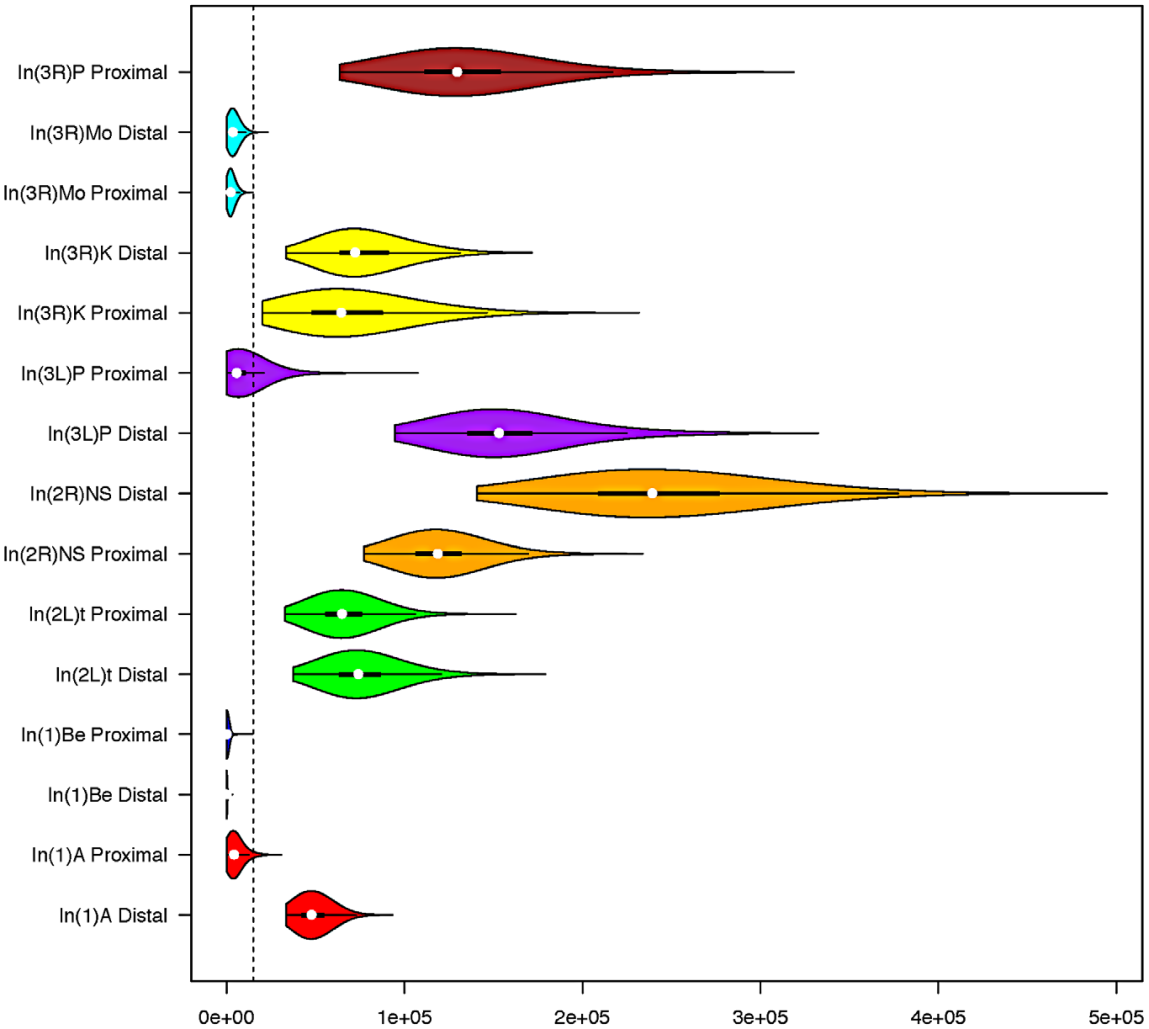
Standard box and whisker plots, with overlaid density kernels depicting the posterior distribution of polymorphism-based age estimates of each inversion breakpoint. Distributions that share a color indicate they are from different breakpoints of the same inversion. The dashed vertical line represents the approximate timing of the out of Africa migration (15,000 years) [22]. In(3R)P's proximal breakpoint is excluded from all analyses because genetic exchange appears extensive and we cannot confidently identify the original haplotype that the inversion captured.

**Figure 3:**
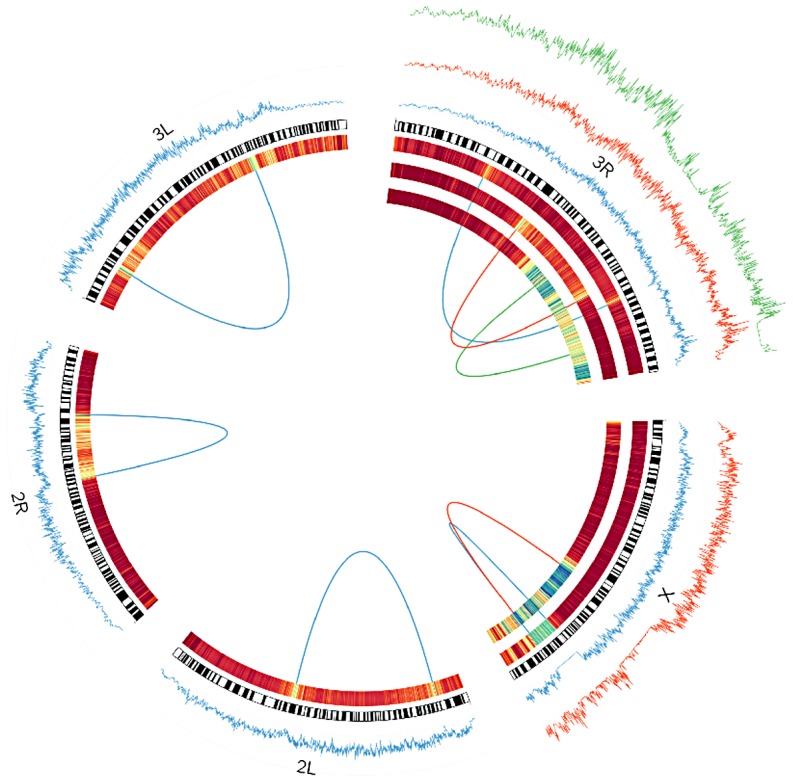
Windowed π among inverted chromosomes (tracks on the outside), and *F*_ST_ between inverted and standard arrangments (expressed as a heatmap on the inside) for each inversion studied as a part of this project. In the heatmap, blue corresponds to F_ST_ of approximately.5. Dark red is zero. Windows are in units of 1000 segregating sites. Breakpoints of each inversion are shown as a line connecting each breakpoint. *In(3R)Mo* is based on samples from the Raleigh, NC population. All others are based on comparisons of inverted and standard haplotypes within African *D. melanogaster* samples. Standard arrangement-bearing haplotypes were filtered for putatively admixed regions identified by Pool *et al*. [19] prior to all analyses. On the X chromosome, *In(1)Be* is shown in blue and *In(1)A* is in red. On 3R, *In(3R)Mo* is in green, *In(3R)K* is in blue and *In(3R)P* is in red. Although the inversions depicted have reversed the order of the segment between breakpoints, this figures displays these regions as collinear to the standard reference sequence for simplicity of comparison.

In two instances (*In(3L)P* and *In(1)A*), the posterior distributions obtained from opposite breakpoints of a single inversion were discordant (Figure 2). One plausible explanation is that by reducing local recombination rates, inversions increase the “range” of genetic hitchhiking [22]. Indeed, the large segment (∼2 MB) of depressed polymorphism surrounding the distal breakpoint of *In(1)A* appears consistent with a hitchhiking explanation (Figure 3). In scans for selection within the Rwandan population, Pool *et al.* [25] found that the sequence corresponding to *In(3L)P*'s centromere-proximal breakpoint is the eighth-ranked genome-wide outlier region for Sweepfinder's [39] *Λ_max_* statistic, which is consistent with recent positive selection associated with this region of the genome. We therefore treat the oldest posterior distribution for each inversion as the better estimate, but we still provide the posterior distribution obtained for the other breakpoint in Figure 2. Although none of the breakpoints, besides *In(3L)P's* proximal breakpoint, are contained within the regions present in the 5% tail of *Λ_max_* distribution, polymorphism at other breakpoints may also be affected by selection on linked sites (which may be very distant in an inversion). As such, a polymorphism-based approach may underestimate inversion ages, and it may be preferable to interpret these results as lower bounds of inversion ages.

Under the assumptions of the exponential-growth model, we find that most inversions are quite young. Median age estimates range from 60 to 239,102 years (95% CI's 5.9–373 and 172,236–336,440 respectively; Figure 2; Table S3), and these estimates are largely consistent with previously work on common cosmopolitan inversions [19], [20], [22]. As might reasonably be expected, all four endemic and rare cosmopolitan inversions appear to be younger than the four common cosmopolitan inversions. Although the majority of research has focused on the common cosmopolitan inversions (*In(2L)t*, *In(2R)NS*, *In(3R)P*, and *In(3L)P*), this suggests that less-studied rarer inversions may prove to be instructive in addressing fundamental questions concerning a novel arrangement's initial increase in frequency.

Based in large part on evidence from *D. melanogaster*, Andolfatto *et al.* [40] observed that polymorphic inversions in *Drosophila* species tend to be young relative to the *T_MRCA_* arrangement from which they are derived. Although our estimates are qualitatively consistent with this observation, particularly among the endemic and rare cosmopolitan inversions, our data suggest that many inversions segregating at moderate frequencies within *D. melanogaster* are significantly younger than previous findings. Although they estimated inversion ages based on a different method, it is noteworthy that Wallace *et al.* [34] found that many of the 3^rd^ chromosome inversions of *D. psuedobscura* are an order of magnitude older than we find for *D. melanogaster*. As noted above, their data also indicate that nucleotide diversity is significantly higher in breakpoint-proximal regions. In short, it is unclear at present if the very young ages of inversions in *D. melanogaster* are a general feature of segregating inversions, or specific to this species.

Undoubtedly, additional examples are needed to definitively address the apparent differences between species. However there is some evidence that the inversions of *D. melanogaster* are unusual with respect to its close relatives. *D. melanogaster* has more than 500 segregating arrangements, some of which are present throughout the species' range, but its sister species, *D. simulans*, harbors no inversions at polymorphic frequencies [10], [41], [42]. A single large paracentric inversion has fixed on the *D. melanogaster* lineage since its last common ancestor with *D. simulans*, and no inversions fixed since the common ancestor with *D. yakuba* approximately 13 million years ago, while the *D. yakuba* lineage acquired 28 inversions during this same timeframe [10], [42].

One plausible explanation for these puzzling observations and the young ages of segregating inversions in this species, is that *D. melanogaster*'s ancestors did not harbor polymorphic inversions, and this species' genome has only recently become tolerant of inversions. This hypothesis was originally proposed in Langley *et al.* [33]. It is unclear why *D. melanogaster* would shift from the ancestral state, which is retained in *D. simulans* and related island endemic species, but the evolution of inversion-tolerance could be expected to leave a detectable signal in the genome. Specifically, genes functionally important for achiasmatic segregation may be expected to show evidence of positive selection specific to *D. melanogaster* or to inversion-tolerant lineages, and we may observe geographically-structured selection associated with populations that contain different frequencies of segregating inversions.

### Geographic Origins

While previous studies [19], [20], [22] have attempted to estimate inversion ages, none has directly considered geographic origins. An analysis such as this may inform our understanding of inversion genealogical histories, and suggest potential selective mechanisms. One feature of *D. melanogaster*'s demographic history is useful in this regard: this species emerged from ancestral African populations and colonized the rest of the world approximately 10,000–15,000 years ago [28], [29]. During this expansion, cosmopolitan populations experienced a sharp bottleneck, which reshaped patterns of nucleotide variation genome-wide. This bottleneck event left a detectable signature in nucleotide data, and it can be used to estimate sub-Saharan *vs.* cosmopolitan origin for specific haplotypes [25]. Using a simple divergence metric, we judge six of the eight inversions studied to be African in origin (*In(2L)t*, *In(2R)NS*, *In(3L)P*, *In(3R)K*, *In(3R)P*, and *In(1)A*), as they are all approximately equally divergent from the African and French sequences and their nearest neighbor is invariably African—this is expected for African haplotypes given the demographic model proposed by ref. [25] (Table S5).

*In(1)Be* and *In(3R)Mo*, which are also the two youngest inversions studied, appear to be two exceptions to the predominantly African origins of inversions. Including the breakpoints, there are four large haplotypes in strong linkage disequilibrium with *In(3R)Mo* (Figure 3) [36]. FR310, the only DPGP2 line that contains *In(3R)Mo*, also contains these haplotypes, and the sequence at each is on average more divergent from African than French lines. Additionally, the least divergent individual haplotype is invariably French. Collectively, these considerations provide strong evidence that *In(3R)Mo* is cosmopolitan in origin; however, note that *In(3R)Mo* is not as closely related to other French genomes as they are to each other (Table S5), suggesting that this inversion may have originated in a different cosmopolitan population.

*In(1)Be* has almost no segregating sites across the entire 1.7 Mb length of the eight samples of this inversion (Figure 3). We find that this haplotype is less divergent on average from French than from African genomes, and that its closest relative at each breakpoint is French, not African (Table S5). This suggests the inversion captured a cosmopolitan haplotype, a conspicuous finding in light of its young age and the fact that this inversion has never been reported outside of Africa even though many cosmopolitan populations have been extensively surveyed [10]. Despite their similar geographic origins, *In(3R)Mo* displays the opposite distribution of *In(1)Be*. *In(3R)Mo* has been identified almost exclusively in cosmopolitan populations, having only been reported from a single South African population [24], which is likely highly admixed [43]. It is interesting to note that introgression patterns of cosmopolitan inversions appear to be the opposite of collinear regions of the genome, in which autosomal chromosomes exhibit significantly more cosmopolitan admixture in Africa than the X chromosome [25].

Predicted geographic origins of inversions agree with and lend further support to the polymorphism-based estimates. That is, both cosmopolitan inversions appear to be younger than the predicted time of the out-of-Africa migration of *D. melanogaster* (Figure 2), which suggests that we have not overestimated inversion origins based on polymorphism. Somewhat more compelling is the observation that at least one of the breakpoints of all African-originated inversions appears to be older than the predicted timing of the out-of-Africa demographic event (Figure 2, Table S4). Although this is not a necessary condition for consistency between these analyses (an inversion could originate on an African haplotype after the cosmopolitan expansions), the fact that we do not observe this pattern suggests that hitchhiking effects may not have drastically affected age estimates at both breakpoints of the same inversion.

The haplotype admixture analysis of [25] sought to resolve sub-Saharan versus cosmopolitan (admixed) ancestry in a panel of African genomes that included chromosome arms bearing inversions. Although the question of recent population ancestry is distinct from that of inversion origin (*e.g.* a haplotype carrying an arrangement that originated in Africa could be considered “cosmopolitan” if it went through the out-of-Africa bottleneck), it may be worthwhile to examine the potential influence of inversions on demographic inferences of this type. Whereas we focus on the comparison of chromosomes with known inversion genotypes, the analysis of Pool *et al.* [25] considers all Rwandan and French genomes as representative of cosmopolitan and African haplotypes regardless of arrangement, and regions around inversion breakpoints are often flagged as admixed by their analysis. This result may be due to powerful effects of inversions on haplotype structure, which is most pronounced when inversions are at different frequencies between populations. Consistent with this explanation, African strains that bear *In(3L)P*, *In(3R)P*, *In(3R)K* and *In(2L)t* (all of which are present in the French population), are often identified as admixed near breakpoint regions. In particular, African *In(3L)P* haplotypes are masked up to approximately 1.5 Mb from the breakpoints by the admixture-detection method of ref. [25]. This could result from this inversion's absence from the Rwandan “African reference” population. Especially in light of their strong population frequency differences and their extended chromosomal influence on diversity (see below), it appears that inversions may strongly impact demographic analyses, potentially resulting in spurious inferences if they are not considered individually.

### Inversions Modify Arm-Wide Patterns of Polymorphism

**Figure 4:**
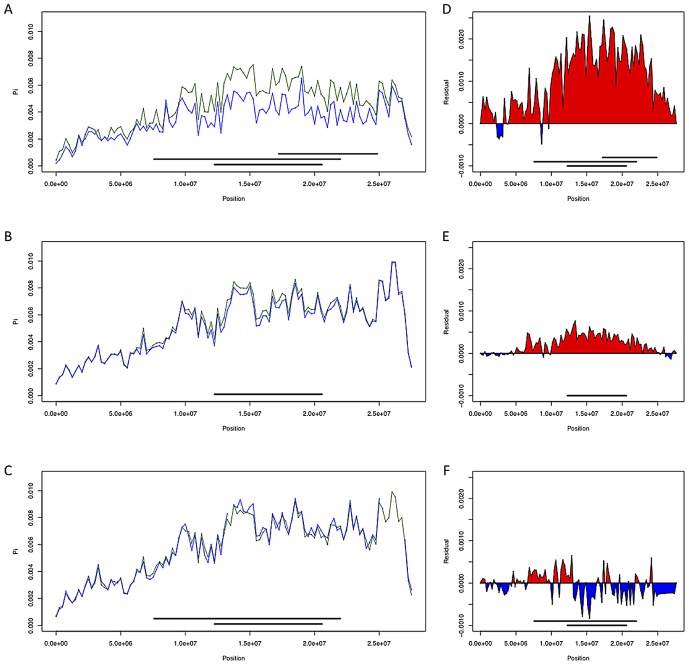
Chromosome arm 3R diversity and residuals with and without inversions. (A–C; France, Rwanda, and Gabon respectively) Chromosome arm 3R diversity (π) in non-overlapping 250 kb windows with inversions included (in green) and without (in blue). (D–F; France, Rwanda, and Gabon respectively) diversity residuals with and without inversions. Red indicates increased diversity relative to standard sequences when including inversions *In(3R)Mo, In(3R)P* and *In(3R)K* while blue indicates decreased diversity with these inversions included.

Given the powerful effect of inversions on nucleotide sequences near to their breakpoints, it is natural to ask whether inversions also affect polymorphism in more distant regions of the chromosome. Neutral models of exchange predict that inversions should be genetically indistinguishable from standard haplotypes towards the middle of inverted regions shortly after achieving equilibrium frequencies [44]. Nonetheless, there are numerous instances in which different chromosome arms within the DPGP2 dataset produce substantially different estimates of nucleotide diversity [25]. In the French population, levels of polymorphism on the autosomal arms correlate with the number of inversions present. Removing lines bearing common inversions sharply reduces this effect (Figure 4, see also [25]).

To investigate the effects of inversions on estimates of polymorphism in additional populations, we compared pairwise nucleotide diversity (π) on chromosome arm 3R in the France, Rwanda, and Gabon populations both including and excluding inversion-bearing haplotypes (Figure 4). In African populations, we observe only a modest effect of inversions on nucleotide diversity. Diversity in Rwanda is slightly increased, likely owing to the low frequency of *In(3R)P* in this population (Table S6); in Gabon nucleotide diversity is slightly reduced, perhaps because of the high frequency of *In(3R)P* in this population and the low diversity within this arrangement. In the French sample, we observe a sizable (∼30% across all of 3R) increase in nucleotide diversity when inversions are included (Figure 4, see also [25]).

**Figure 5:**
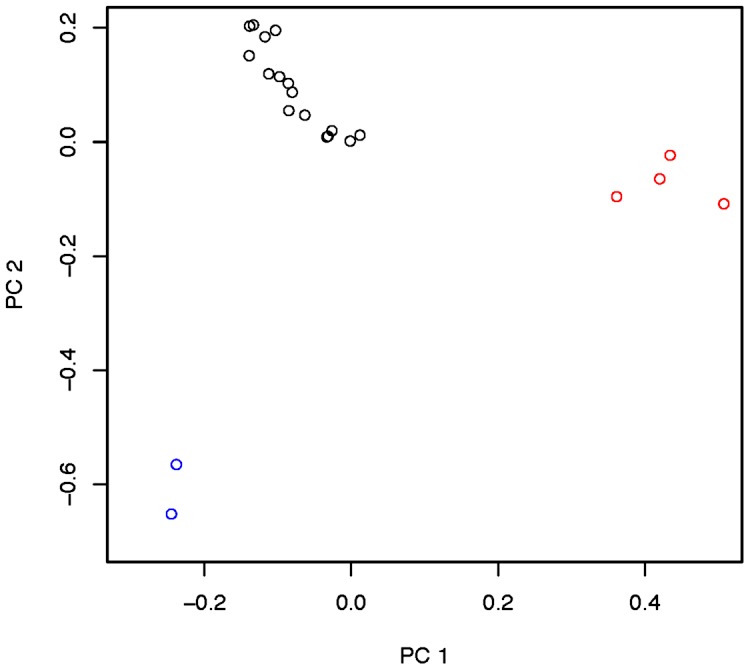
Principle components 1 and 2 within the Rwandan (RG) population with admixture-tracks and centromere and telomere proximal segments. Described in Pool *et al*. [19], masked. Red indicates *In(3R)P* bearing lines. Blue indicates *In(2L)t* bearing lines, and black indicates lines which bear the standard arrangement for both inversions. There are no lines in this sample that harbor both *In(3R)P* and *In(2L)t*.

**Figure 6:**
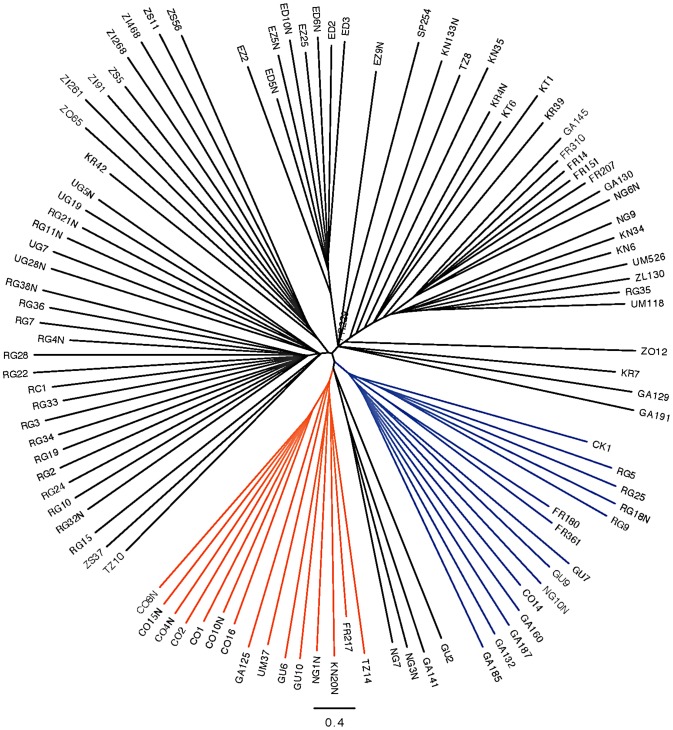
Neighbor joining tree of all “primary core” samples using only chromosome arm 3R. *In(3R)K* branches are labeled in orange and *In(3R)P* is in blue. All other clustering appears to be largely geographic and clustering with arrangements is also largely geographic.

Inversion-mediated effects on nucleotide diversity may be pertinent on a genome-wide scale as well. Principle component analysis, as described in [25] and following the method of [45], within the Rwandan samples suggests that inversions are responsible for the majority of genetic structure in this population (Figure 5). Importantly, this does not appear to be limited to breakpoint regions, instead inversions affect polymorphism through the majority of chromosome arms (Figure 4). Consistent with previous work in *D. pseudoobscura* and related species [46], we observed increased differentiation between arrangements in regions up to 4 Mb outside inversion breakpoints (Figure 4). Note also that inversion-bearing chromosome arms are more closely related to individuals that have the same arrangement in other populations than to standard haplotypes within their own population (Figure 6).

It is known that recombination in heterokarytopyes within inverted regions is infrequent [4], [8], [10], [47]. Existing estimates in *Drosophila* suggest a neutral recombination rate of approximately 10^−4^ for double recombination events towards the center of inversions [47], and theoretical predictions suggest exchange rates may be as large as 10^−2^ in the center of large inversions [44], [48]. As the inversions studied here are young, some level of differentiation may be attributable to their unique origins and suppressed recombination. Still, even low rates of exchange are expected to rapidly eliminate genetic differentiation between arrangements [44], [48].

A likely explanation of differential diversity associated with inversion-bearing haplotypes is that inversions migrate at different rates between populations than standard haplotypes. In particular, arm 3R inversions, especially *In(3R)P* and *In(3R)K*, increase diversity by ∼30% in the French population (Figure 4). Because different cosmopolitan populations typically contain similar sets of genetic variants [49], it seems likely that most inversion bearing haplotypes present in the French sample are recent migrants from African or African-admixed populations. As described above, chromosome arms with fewer or no inverted haplotypes in this sample show concordant decreases in polymorphism, which supports a differential-migration interpretation. However the evolutionary drivers of inversion introgression remain largely unknown.

### Patterns of Nucleotide Variation Are Consistent with Selection

**Figure 7:**
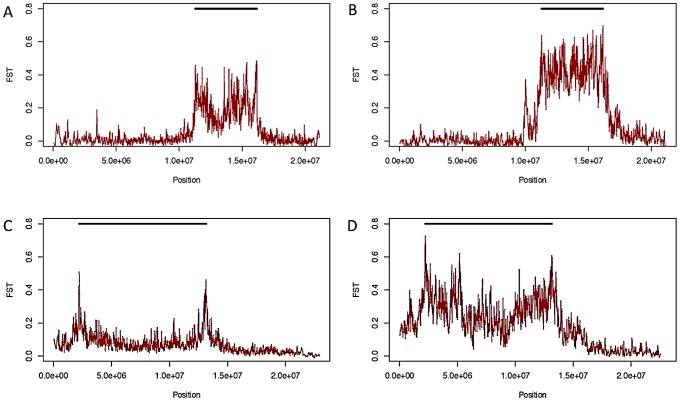
*F_ST_* between standard and inverted haplotypes. *F_ST_* between standard and inverted haplotypes for *In(2R)NS* in African samples (A) and samples from Raleigh, NC (B), and for *In(2L)t* in African (C) and Raleigh (D) samples.

Although inversions retain some genetic differentiation from standard haplotypes, in African samples *F_ST_* decays quickly with increasing distance from breakpoints in all inversions except *In(1)Be* (Figure 3); thus we expect it will soon be possible to test hypotheses regarding the selective maintenance of co-adapted alleles (reviewed in [4], [8]). Because we lack specific knowledge of neutral recombination rates in heterkaryotypes and population demographic models, and because the African sampling is distributed among many geographically diverse populations, we do not feel these data are suitable for a rigorous quantitative test of these selective hypotheses. Nonetheless, we do note numerous regions of decreased polymorphism and strong genetic differentiation between arrangements that are qualitatively suggestive of selective mechanisms in many inversions (Figure 3, Figure 7).

As noted in [33], *In(3R)Mo*, which is present in ∼12% of the strains in the DGRP sample, is in strong linkage disequilibrium with two large haplotypes that are not immediately adjacent to inversion breakpoints. One of these haplotypes lies outside the inversion, between the distal breakpoint and telomere (Figure 3). These haplotypes are shared with the single *In(3R)Mo* bearing line in the DPGP2 dataset. Thus this pattern of long-range linkage disequilibrium is not limited to the Raleigh population and may instead be a geographically widespread phenomenon. *In(1)A* displays a similar pattern in the regions surrounding the distal breakpoint, with numerous haplotypes in strong linkage disequilibrium with the inversion (Figure 3). It is not clear whether these and other conspicuous patterns of variation are consistent with the maintenance of co-adapted alleles [e.g. 4], [50] or selective sweeps [51] specific to one arrangement [33]. This important distinction is in some ways an extension of the ongoing debated between the relative prevalence of background [52] and positive selection [51] and demands further analysis updated with information of the rates of exchange of specific inversion heterokaryotypes.

Likely resulting from the out-of-African bottleneck, the data show that inversions tend to be more genetically differentiated from the standard arrangement in cosmopolitan populations (Figure 7). It therefore appears that the ancestral African populations would be the best suited for fine-mapping alleles that are associated with alternative arrangements via differential selection, and future research in this field should concentrate on samples derived from this region.

### Inversion Breakpoints Rarely Interrupt Genic Sequences

Although the hypothesis has received less attention in the literature, inversion breakpoint mutations may also be the target of selection that affects their evolutionary outcomes [21]. We find that few inversion breakpoints disrupt genic sequences and associated regulatory regions (Table S7). Of the three inversions with simple cut-and-paste breakpoints (see [42] for a description of inversion breakpoint structures), *In(3R)Mo* has one breakpoint situated in an exon, and *In(2L)t*'s distal breakpoint truncates the 3′ untranslated region of CG15387. Many of the inversions with inverted duplications at each breakpoint also interrupt transcribed sequences. In most cases, the duplicated portion may retain an intact copy (*e.g. In(3R)P* [20]). However, two inverted-duplication bearing inversions, *In(1)A* and *In(1)Be*, have both breakpoints of the duplicated regions situated in single genes (Table S7). Thus, four inversions breakpoints may have produced structural or regulatory changes in genic sequences. *In(3L)P*'s distal breakpoint also interrupts a transcribed cDNA [21], but presently there are no annotated transcripts that correspond to this region.

While it is possible that breakpoint mutations are the selective mechanisms by which inversions achieve polymorphic frequencies [21], we favor a model in which these effects are deleterious byproducts of the inversion formation. The proportion of breakpoints that interrupt genic sequences is significantly fewer than expected if we assume breakpoints form randomly with respect to genic sequences and uniformly across chromosome arms (*P* = 0.000122; Permutation Test). Furthermore, two studies that focused on inversion fixations between *Drosophila* lineages do not report interrupted genic sequences associated with inversion breakpoints [42], [53]. Thus, breakpoint mutations may often oppose inversions' fixation in natural populations. Deleterious consequences of interrupted genic sequences cannot be too severe, since all of the inversions that we studied are regularly homozygous in phenotypically normal isofemale lines, and those situated on the X chromosome must often exist in hemizygous states in natural populations.

### In(1)Be Increases Transmission via Sex-Ratio Distortion

To this point, we have characterized inversion genealogical histories, and presented evidence that selective mechanisms affect their distributions. Of course, it would be of interest to identify specific sources of natural selection that affect the distributions of inversions. That *In(1)Be* arose on a cosmopolitan haplotype and is currently invading African populations suggests that this inversion may harbor a sex-ratio distortion complex. Though there are no known cases from *D. melanogaster*, X chromosome inversions in other *Drosophila* species are commonly associated with sex-ratio distortion [6]. There is prior evidence that cosmopolitan X chromosomes from this species can drive against African Y chromosomes [54]. Hence, a wealth of background information, in combination with suggestive population genetic signatures, prompted us to investigate sex-ratio distortion as one potential mechanism influencing *In(1)Be*.

In seven of the eleven experimental crosses, we find significant evidence for distortion at the α = 0.05 level and in all crosses the average sex ratio trended towards females. Three of the experimental crosses remain significant after applying a Bonferoni correction for multiple testing. None of the control crosses, which are derived from many of the same populations as *In(1)Be* bearing strains, show significant evidence for a transmission bias (Table 3). It is formally possible that this inversion does not drive, but that the test lines we selected also happen to contain a segregation distortion complex outside of the inversion. Because it is polymorphic for this inversion, strain RG11N is a more definite control. We find that that male progeny from RG11N mothers that inherit *In(1)Be* transmit their X chromosome at higher than Mendelian expectations, while those that inherit the standard arrangement do not (Table 3). To exclude differential viability as an explanation, we counted all eggs laid by females mated to RG11N(*In(1)Be*)/ZS30(Y) F_1_ males. Even when we conservatively assume that all preadult mortality is suffered by males, we find a significant excess of female offspring (*P* = 0.0248; Table 3).

**Table 3:** Summary of data from crosses testing for sex-ratio distortion in *In(1)Be* bearing males.

X Chromsome	Y Chromsome	F_1_ males	Progeny	K	p			
GA191N	ZS30	9	1451	0.538	0.001933*			
GA191N	C5	5	355	0.563	0.009701			
GA191N	C17	4	344	0.547	0.037524			
GA191N	K12	5	489	0.517	0.234691			
RG10	ZS53	5	751	0.543	0.009731			
RG10	C5	4	379	0.531	0.108800			
KR39	K12	5	567	0.526	0.103836			
KR39	C17	5	457	0.565	0.002475*			
RG11N	C17	5	417	0.52	0.216678			
RG11N	K12	4	221	0.557	0.040032	No. Eggs Laid	K**	P**
RG11N	ZS30	18	2640	0.541	0.000012*	2724	0.524	0.005406
	Average	6.27	733.72	0.541				
Control Crosses:								
RG11N∧	ZS30	12	1305	0.508	0.289922			
RG22	C17	5	478	0.52	0.192423			
RG22	ZS53	4	358	0.495	0.562976			
RG22	C5	5	523	0.504	0.430587			
RG35	ZS30	5	704	0.521	0.137196			
RG35	K12	4	229	0.546	0.072919			
KR42	K12	5	400	0.509	0.363193			
KR42	C5	5	380	0.483	0.747551			
KR42	C17	5	444	0.511	0.334670			
	Average	5.56	535.67	0.511				

K is the proportion of females emerging from each cross.

* remains significant after applying a bonferoni correction for multiple testing.

** K and p after conservatively assuming all pre-adult mortality is suffered by males.

∧ RG11N is listed as a control and experimental cross because it is polymorphic for *In(1)Be*.

It is plausible that *In(1)Be*'s recent rise in frequency is due to selection favoring this transmission bias. The strength of drive (*k*∼0.541), though weak by comparison to other sex-ratio distortion systems in many other *Drosophila* species (reviewed in [6]), is substantial when compared to most selection coefficients in the genome and is similar to an existing estimate of drive strength of cosmopolitan X chromosomes against African backgrounds (*k*∼0.61) [54]. *In(1)Be* is within the 95% confidence interval of map location for this complex, though this interval is very wide [54]. The *Recovery Disrupter* sex-ratio distortion complex, which may be the same as [54] studied, has been mapped more precisely to cytological band 1–62.9, and has been suggested to act in natural populations [55], [56]. This cytological band also contains the proximal breakpoints of both *In(1)Be* and *In(1)A*.

It is possible that a locus near band 1–62.9 may have recurrently evolved drive during the recent evolutionary history of this species, and has acquired at least one recombination-suppressing inversion. This may explain the extreme proximity (816 bp) of *In(1)Be* and *In(1)A*'s proximal breakpoints (but we note that breakpoint reuse may result from neutral mechanisms [42]). In pilot crosses, we did not observe sex-ratio distortion associated with *In(1)A* (not shown). Since this inversion is considerably older than *In(1)Be*, suppressors specific to the driving allele captured by *In(1)A* may have achieved high frequencies, effectively masking distortion associated with *In(1)A*. Similar results have been inferred in other sex-ratio distortion systems in *Drosophila* (reviewed in [6]). Testing *In(1)A* for distortion on a wider range of African and cosmopolitan lines is a target of future research.

## Conclusion and Prospects

The majority of existing work on inversions has focused on well-established polymorphisms. Young inversions provide a valuable counterpoint because they yield a glimpse of the mechanisms that lead to their initial rise in frequency. The forces involved in the initial rise need not be the same as the ones involved in long-term maintenance of inversions; examples from young inversions are essential to addressing this potential difference. In the case of *In(1)Be*, we have identified a likely mechanism for this inversion's rapid increase in frequency, namely, sex-ratio distortion. Young, rare inversions are also commonly associated with *Segregation distortion* haplotypes in this species [5] including one that is currently sweeping African populations [7], suggesting that distortion may be a common means by which inversions initially achieve high frequencies. Therefore, testing inversions for segregation distortion may be a fruitful approach. Finally, for *In(3R)Mo*, the linked-haplotypes found outside of the breakpoints also make attractive targets for genetic dissection.

Numerous models of inversion evolution posit that selection favors different alleles in alternative arrangements. Because they will be more amenable to surveys via population genetic modeling, older inversions of *D. melanogaster* provide an ideal system to test these hypotheses. That is, because recombination has had more time to decouple selective and neutral processes, older inversions should afford better resolution of specific alleles in linkage disequilibrium with inversions. Though importantly, our data suggest that sampling should be focused on ancestral African populations where selective and demographic/neutral patterns of variation may be more easily distinguished. Though low levels of genetic differentiation remain between arrangements, we observe rapid decay of genetic differentiation with increasing distance from breakpoints. Hence, in combination with neutral estimates of recombination in heterokayotypes, it will soon be possible to test widely popular selective hypotheses that suggest inversions achieve high frequencies via maintaining linkage disequilibrium between co-adapted alleles.

One important message of this work is that inversion data should be interpreted with caution. Inversions interact powerfully with diversity in the sequences immediately proximal to their breakpoints and chromosome wide. Failure to account for inversions may lead to spurious results (nonetheless, it is probably wise to exclude these regions from population genetic analyses that assume normal recombination). As we have shown, inversions also have diffuse effects on polymorphism, which may further complicate demographic modeling by producing substantial arm-specific effects. Thus, even population genomic studies that are not focused specifically on inversions cannot ignore their presence in the data. The reverse is also true; population genetic analyses focused on inversions may be affected by sampling if the recent demographic history of the species is not explicitly considered.

Far from being a nuisance in the analysis of population genomic data, inversions may prove fertile ground for the study of genome evolution and mechanisms of selection. We hope that our analysis will help reignite interest in naturally occurring inversions. Although studied extensively for almost a century, little progress has been made towards conclusively understanding the selection that affects inversion polymorphisms. With the increasing availability of genomic techniques, it is now possible to reopen many longstanding questions. In combination with an exceedingly well-curated reference genomes and an enormous body of literature, these bioinformatic and computational tools make the inversion polymorphisms of *D. melanogaster* an appealing model system once more.

## Methods

### Sequence Data

The majority of analyses in this study are focused on sequence data from second sequencing phase of the *Drosophila* Population Genomics Project (hereafter DPGP2; www.dpgp.org). See [25] for a description of this primary data set, which was the source of all African and European samples. We limited most our analyses to the target, ‘core’ genomes described in [25]; although we included all strains that contained inverted haplotypes regardless of core or addendum status in breakpoint analyses. To study inversions from one cosmopolitan population, we downloaded assemblies [26] and short read data (NCBI SRTA) for the *Drosophila* genetic resource panel (DGRP), which is derived from a population from Raleigh, NC. For analyses of breakpoint regions, we apply RAR to generate unbiased sequence data. Because the dataset produced by Mackay *et al.* [26] is comprised of numerous sequencing technologies, we restricted breakpoint analyses to lines that had been sequenced with illumina paired-end reads. Analyses focused on comparative polymorphism across chromosome arms, such as windowed π and *F_ST_* analyses, rely on the assemblies produced by [25] and [26].

Because Mackay *et al.* [26] did not mask regions that fail to inbreed, their assemblies contain substantial residual heterozygosity. These regions are obvious under cursory inspection [23], [33], so we masked all chromosome arms that demonstrated long tracks (identified using 1/2 mb windows) of residual heterozygosity from all analyses.

### Inversion Genotypes

We generated inversion genotypes for each line by including the breakpoint-spanning contigs produced by Corbett-Detig *et al.* [23] with the standard *D. melanogaster* reference sequence during initial mapping. Reads overlapping an inversion breakpoint by more than 20 bp were considered evidence that the stock bears the inversion. In all cases, there is a perfect correspondence between breakpoint genotypes, mate-pairs that span an inversion breakpoint (where applicable), the inversion validations performed by [33], and with our unpublished data. See Table S6 for a list of inversion genotypes in each line in DPGP2. See supplemental tables S8, S9, and S10 for inversion genotypes for *In(2L)t*, *In(2R)NS* and *In(3R)Mo* respectively in the DGRP lines. Results are provided in this format due to the presence of residual heterozygosity in DGRP sequence data. That is, we only report individuals that appear to be fixed for the inversion of interest, but do not want to give the impression that other lines may not also contain these inversions. We do not report other inversions, which are surely present in DGRP, because we did not attempt to validate any of these genotypes due to small sample sizes in that panel. See [33] for many PCR-validated inversion genotypes in lines derived from this panel.

### RAR Sequence Production

We aligned all short-read data to the *D. melanogaster* reference genome v5.31 [57] using BWA v0.5.9 [30]. We extracted all read-pairs if either read aligned within 500 bp of a region of interest, and *de novo* assembled all reads for each region using PHRAP v1.090518 (http://www.phrap.org/phredphrap/phrap.html). PHRAP command line parameters used were ‘-forcelevel 10’, ‘-minmatch 15’, ‘-vectorbound 0’, and ‘-ace’. We converted these ‘.ace’ assemblies to SAM format using custom perl scripts, and generated a consensus from the resulting alignment using samtools [31], where we required a minimum depth of 3 and a minimum nominal quality of 50. Finally, we extracted sequences of interest by using cross_match v1.090518 (http://www.phrap.org/phredphrap/general.html) to align the corresponding region from the reference genome to the consensus. We required a minimum alignment length of 100 bp (Figure S1).

### Empirical Validation of RAR

Three of the strains (ZK84, ZK131, and ZK186) that were resequenced as a part of DPGP2 have been studied extensively via Sanger-PCR sequencing [27], [29] (EMBL ascension numbers are provided in Table S1). For each, there is more than 50 kb of high quality X-chromosome sequence data available. This population is among the most diverse analyzed for this project or ever identified in the species. In addition, these sequences are primarily derived from intergenic regions, and are therefore a conservatively challenging test for RAR's performance.

Using RAR, we assembled sequences corresponding to each available PCR fragment in each line. We resequenced via PCR those fragments where we identified mismatches between the RAR consensus and PCR-derived sequences. All PCR was performed on the original DNA extraction used for library preparation. The generated PCR traces were aligned to the original EMBL sequence and to the RAR assembly using clustalW version 2 [58]. We also experimented with additional iterations (*i.e.* replacing the reference with corresponding RAR contigs and realigning all reads to this augmented reference sequence), but observed little improvement relative to a single reassembly (not shown).

To investigate the effect of decreasing read depth, we reran RAR after randomly discarding 10 to 90 percent of the reads (in 10% intervals), on these same regions. We performed 100 bootstrap replicates at each proportion of reads discarded for each line. The resulting contigs were then aligned to both the Sanger-PCR sequences as well as the reference, and their divergence from each recorded.

We assembled sequences for each line immediately inside each inversion's breakpoints using RAR. Alignments were performed using clustalW version 2 [58]. All alignments were inspected with assistance from PERL scripts, which we designed to flag problematic regions surrounding indels and SNPs shared between inverted and standard arrangements. Multiple alignments for all SNPs within 10 bases of an indel and all shared polymorphism was inspected manually. In all but two cases, shared polymorphisms were present on an inverted haplotype flanked with other shared polymorphisms. This is an expected signature of genetic exchange between arrangements, and we masked all sequences that we inferred resulted from recombination between inverted and standard arrangements. Finally, we estimated local rates of mutation by aligning the reference sequence from regions we used for demographic analyses with a recently improved *D. simulans* reference genome [59].

### Sequence Selection and Processing

At In(3R)P's distal breakpoint, genetic exchange with the standard arrangement has been extensive, and we could not confidently determine which samples retained the original haplotype that the inversion arose on. We therefore excluded this breakpoint from all subsequent analyses. For all other breakpoints, we were able to infer the original haplotype captured by the inversion event, and we discarded all recombinant haplotypes from downstream analyses.

### Inversion Age Estimates

Empirical estimates of summary statistics, specifically *π*, θ_W_, Tajima's D, and D′, were obtained by treating all missing data and indels as complete deletions. Simulations were performed in ms [60] following a rejection-sampling approximate Bayesian computation approach. Briefly, we modeled each inversion as a population that has grown exponentially at the same rate since its formation through the present. We calculated the expected theta of the current inverted population as:
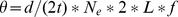
Where L is the total length of the aligned sequences. d is the nucleotide divergence per site between *D. simulans* and *D. melanogaster* per site. t is an approximation of the time of divergence between these two species in generations (30 million). N_e_ is a widely used estimate of the *D. melanogaster* effective population size (10^6^), and f is the frequency of the inversion in the DPGP2 or DGRP datasets as appropriate.

To accommodate uncertainty in our estimate of 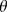, we selected values for simulations from uniform(0, 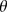 *10). We defined the tolerance for the number of segregating sites and π as no more than 5% different from empirically obtained data from the sequence alignments. We stored alpha, theta, and *T_MRCA_* of the sample for each accepted simulation, and ran each until at least 10,000 simulations were accepted for each inversion breakpoint. We obtained a posterior distribution of estimates for the age of each inversion as:
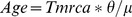
Where 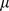 is in units of substitutions per region per year, and T_MRCA_ is in units of 4N_0_. Thus this equation yields a distribution of the estimates of the age of the inversion in years.

### Geographic Origins

To estimate geographic origins, we compared breakpoints regions of each inversion with both the cosmopolitan (FR) and African (RG) populations. We noted both the average divergence to lines that bear the standard haplotypes in the French population and African populations, as well as the nearest neighbor. We judged each inversion as cosmopolitan if both the nearest neighbor was a standard French sequence, and the average divergence between the French population and this inversion was less than the average divergence from African sequences.

### Experimental Crosses

We initially tested the eight DPGP2 strains identified as carrying *In(1)Be* for fixation of the inversion using PCR primers we have developed (unpublished work). We identified three strains, GA191N, KR39, and RG10 that have fixed In(1)Be. RG11N is segregating for this arrangement. For control crosses, we selected three strains from the same populations, RG22, RG35, and KR42, and we confirmed that each is fixed for the standard X chromosome arrangement. Virgin females from these stocks were crossed to five strains, which are known from previous work to be susceptible to cosmopolitan sex-ratio distortion, K12, C5, C17, ZS30, and ZS53 [54]. The resulting male progeny were then crossed individually to two virgin Oregon-R females, aged 3 to 8 days. All crosses were performed in vials on standard corn-meal medium supplemented with yeast and maintained at 25°C. After three days we discarded the parents. We counted male and female offspring each day after the first flies emerged until 15 days after removing the parents. Crosses to test for differential viability as an explanation of the observed sex-ratios were performed identically, except that parents were flipped to a new vial every eight hours during the laying period, and we counted eggs immediately afterwards.

### Genomic Polymorphism Analyses

Windowed summary statistics for inverted and standard populations were calculated based on the assemblies produced by Pool *et al.* [25] and Mackay *et al.* [26]. We masked all putatively heterozygous sites prior to this analysis. In both datasets, approximately 1% of non-reference alleles are heterozygous outside of residually heterozygous regions. Although this is a relatively small proportion, this practice of excluding heterozygous sites may be dangerous in serious quantitative analyses; however, for our purposes, which are largely oriented towards qualitative, broadscale observations, this is unlikely to present a major issue. We calculated *π* without applying any sampling thresholds. We calculated *F_ST_* as described in [61], excepting that we did not weight polymorphism estimates by the sample size.

## Supporting Information

Dataset S1 Alignments of new Sanger-sequencing traces for all sequences containing a mismatch with RAR sequences.
(ZIP)

Figure S1 RAR Workflow. Reads are initially mapped to a reference genome. Reads that map, or whose pairs map, to a region of interest are parsed and *de novo* assembled used PHRAP (http://www.phrap.org/phredphrap/phrap.html). Clones for which only one read in a pair mapped initially are shown in green.
(TIF)

Table S1 EMBL accension numbers used in RAR empirical confirmation.
(TXT)

Table S2 Inversion breakpoint summary information for divergence-based aged estimates. In(1)Be and In(3R)Mo are not included because they are cosmopolitan in origin and therefore almost certainly younger than 15,000 years old.
(TXT)

Table S3 Summary statistics for single nucleotide polymorphism data derived from RAR assemblies of intergenic regions immediately inside inversion breakpoints. Regions of exchange between standard and inverted regions were removed prior to all calculations.
(XLS)

Table S4 95% credible interval and median ages estimated via polymorphism within the inverted haplotypes at each breakpoint.
(XLS)

Table S5 Average genetic distance of each inverted haplotype at breakpoints in comparison with French and Rwandan genomes. All standard-haplotype Rwandan genomes were masked for admixture, as identified by Pool et al. [25], prior to this analysis.
(XLS)

Table S6 Inversion genotype calls for each strain in DPGP2. Genotypes are only provided for target chromsomes (column 2).
(XLS)

Table S7 Summary of inversion breakpoint structure. Gene's interrupted is included for any ‘cut-and-paste’ inversion breakpoint that is situated in annotated genic regions, but is only listed for ‘inverted duplication’ inversion breakpoints if both breakpoints interrupt the same transcribed sequence as described in the main text.
(XLS)

Table S8 *In(2L)t* homozygous lines in the DGRP.
(TXT)

Table S9 *In(3R)Mo* homozygous lines in the DGRP.
(TXT)

Table S10 *In(2R)NS* homozygous lines in the DGRP.
(TXT)
