# Correction: Population Genomics of Inversion Polymorphisms in *Drosophila melanogaster*

**DOI:** 10.1371/annotation/b1cace11-17ed-456e-b8a9-006c09125bd0

**Published:** 2013-08-16

**Authors:** Russell B. Corbett-Detig, Daniel L. Hartl

Tables S9 and S10 were incorrectly switched. The file that appears as Table S9 should be Table S10, and the file that appears as Table S10 should be Table S9. The table legends are in the correct order.

