# Supplementary figures and images for: Population Genomics of Inversion Polymorphisms in Drosophila melanogaster
Source: PLoS Genet. 2012 Dec 20;8(12):e1003056. doi: 10.1371/journal.pgen.1003056 (PMC3527211; doi:10.1371/journal.pgen.1003056)

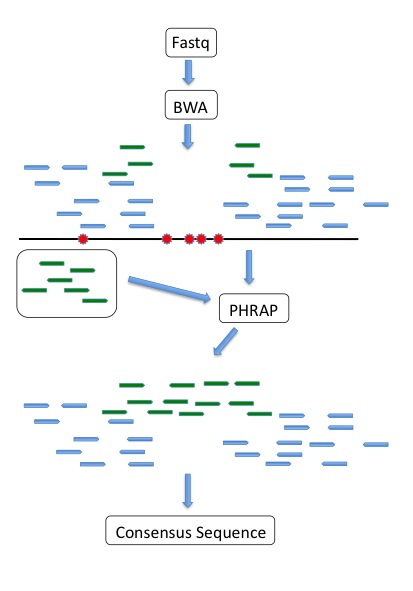

Supplement: Figure S1 — RAR Workflow. Reads are initially mapped to a reference genome. Reads that map, or whose pairs map, to a region of interest are parsed and de novo assembled used PHRAP (http://www.phrap.org/phredphrap/phrap.html). Clones for which only one read in a pair mapped initially are shown in green. (TIF) [file pgen.1003056.s002.tif]
